# Bacterial Community Profiling of Milk Samples as a Means to Understand Culture-Negative Bovine Clinical Mastitis

**DOI:** 10.1371/journal.pone.0061959

**Published:** 2013-04-25

**Authors:** Joanna S. Kuehn, Patrick J. Gorden, Daniel Munro, Ruichen Rong, Qunfeng Dong, Paul J. Plummer, Chong Wang, Gregory J. Phillips

**Affiliations:** 1 Department of Veterinary Microbiology and Preventive Medicine, College of Veterinary Medicine, Iowa State University, Ames, Iowa, United States of America; 2 Department of Veterinary Diagnostics and Production Animal Medicine, College of Veterinary Medicine, Iowa State University, Ames, Iowa, United States of America; 3 Department of Biological Sciences, University of North Texas, Denton, Texas, United States of America; 4 Department of Computer Science and Engineering, University of North Texas, Denton, Texas, United States of America; University Medical Center Utrecht, The Netherlands

## Abstract

Inflammation and infection of bovine mammary glands, commonly known as mastitis, imposes significant losses each year in the dairy industry worldwide. While several different bacterial species have been identified as causative agents of mastitis, many clinical mastitis cases remain culture negative, even after enrichment for bacterial growth. To understand the basis for this increasingly common phenomenon, the composition of bacterial communities from milk samples was analyzed using culture independent pyrosequencing of amplicons of 16S ribosomal RNA genes (16S rDNA). Comparisons were made of the microbial community composition of culture negative milk samples from mastitic quarters with that of non-mastitic quarters from the same animals. Genomic DNA from culture-negative clinical and healthy quarter sample pairs was isolated, and amplicon libraries were prepared using indexed primers specific to the V1–V2 region of bacterial 16S rRNA genes and sequenced using the Roche 454 GS FLX with titanium chemistry. Evaluation of the taxonomic composition of these samples revealed significant differences in the microbiota in milk from mastitic and healthy quarters. Statistical analysis identified seven bacterial genera that may be mainly responsible for the observed microbial community differences between mastitic and healthy quarters. Collectively, these results provide evidence that cases of culture negative mastitis can be associated with bacterial species that may be present below culture detection thresholds used here. The application of culture-independent bacterial community profiling represents a powerful approach to understand long-standing questions in animal health and disease.

## Introduction

Bovine mastitis resulting from an infectious agent is a significant disease in the dairy industry. As a result of decreased milk production, decreased milk quality resulting in lost premiums, and treatment expenses, clinical mastitis cases can cost between $95.31 and $211.03 per case; with an estimated cost to the U.S. dairy industry of approximately $1.7–2 billion dollars annually [1], [2]. Mastitis can be caused by a variety of bacterial pathogens, most commonly coagulase positive and negative *Staphylococcus*, species of *Streptococcus*, and Gram negative bacteria including *Escherichia coli* [3]. However, approximately 10–40% of clinical mastitis cases yield “no significant growth” in routine clinical culture assays, and one study has also indicated that the number of such cases may be on the rise, although the reason for this is not currently known [4]. The lack of identification of microorganisms in culture negative, clinical mastitis cases may have multiple explanations including our inability to culture the bacteria responsible for disease, their presence below current detection thresholds, the absence of the bacteria at the time culture is initiated, or that the mastitis may be caused by non-bacterial microorganisms.

Identifying the microorganisms responsible for culture negative, clinical mastitis and assessing changes in bacterial populations throughout infection will improve our understanding of the disease process allowing us to identify more effective intervention strategies. We further reasoned that application of culture-independent metagenomic approaches would provide new insight into the composition of the bacterial communities associated with culture-negative mastitis. Here we report the use of pyrosequencing of PCR amplicons representing specific regions of 16S rRNA genes (rDNA) directly from milk to characterize the microbiota of “culture negative” clinical mastitis samples. For sequencing, we targeted the V1–V2 hypervariable regions of 16S rDNA. This region has been shown to accurately differentiate between bacterial genera and has been used in 16S rRNA gene studies of samples from mammalian hosts [5]–[7]. Samples were subjected to Roche 454 pyrosequencing with Titanium chemistry, an approach that has been shown to be of sufficient accuracy for identification of bacterial genera based on their 16S rDNA hypervariable regions and offers greater sample depth at a much lower cost than Sanger sequencing [8].

For our study, we collected milk samples from three different dairy farms from cattle exhibiting acute signs of clinical mastitis [9]. From these animals one sample was collected from each quarter and the severity of the clinical case was scored based on physical appearance of the milk and udder and whether the cow was exhibiting signs of systemic disease [10]. The milk samples were then tested for bacterial growth to identify pairs of samples from the same animal with no significant growth but where one sample represented milk from a healthy quarter and the other a mastitic quarter. Milk from cows with no signs of mastitis in any quarter, and low somatic cell counts (LSCC) was also collected and tested for comparison. After isolating DNA from the milk samples, 16S rDNA amplicon libraries were generated and sequenced with Roche 454 pyrosequencing technology. Following sequence data processing, including 16S rDNA sequence classification, members of the microbiota and their relative abundances were examined. Further statistical analyses to evaluate and compare the milk sample microbial compositions were subsequently performed.

## Results

### Milk Collection and Growth Testing

To identify suitable samples for analysis, milk was to be collected from each quarter from 159 mastitic cows for a total of 636 potential samples. However, sample sets from 5 animals plus 1 individual sample were discarded due to fecal contamination of the milk sample, and 10 cows had 14 quarters that were dead and produced no milk. All other samples were tested for bacterial growth for a total of 601 milk samples from 154 cows subjected to screening. Under standard growth conditions, pathogens were detected in samples from 194 quarters (32.3% of screened) of 122 cows (79.2% of screened) and were identified as follows: Coagulase-negative *Staphylococci* in 56 cows (36.6% of infected quarters); *S. aureus* in 14 cows (8.4% of infected quarters); environmental *Streptococcus* in 32 cows (17.5%); *E. coli* in 32 (17.5%); *Klebsiella* spp. in 12 (7.2%); Gram-negative non-coliform rods in 5 (2.6%); Coliform bacteria in 4 cows (2.1%); *Trueperella pyogenes* in 5 cows (3.1% of infected quarters), and *Corynebacterium bovis* in 4 cows (2.6%). Two or fewer cows were also found to be infected with *Serratia* spp. in 3 cows (1.0% of infected quarters); *Bacillus* spp. in 2 cows (1.0%); yeast in 2 cows (1.0%); *Pasteurella multocida* in 2 cows (1%); *Streptococcus* spp. in 1 cow (1.0%); unidentified bacteria in 1 cow (0.5% of infected quarters), and finally, gram-negative non-coliform bacteria in 1 cow (0.5%). Multiple microorganisms were detected in 45 cows, 7 of which had multiple organisms detectable in one or more quarters. No significant growth was detected in 258 of the 601 samples tested (42.9%). Following enrichment for bacterial growth for the 153 mastitic samples collected, 43 (28.1%) of the mastitis samples yielded no significant growth in the clinical mastitis quarters. From these, 26 pairs of samples were identified for which culture negative pairs of mastitis and healthy quarter samples were available for screening. Two LSCC samples that were culture negative following enrichment and 10 sample pairs were selected to obtain a higher sequencing depth per sample (Table S1).

### DNA Isolation and Sequencing Preparation

The total genomic DNA isolated from all culture negative, clinical mastitis samples and one healthy quarter sample was visible by agarose gel electrophoresis. The visibility of DNA from mastitis samples corresponded to DNA yields (3.7–501 ng/µl) that were much higher than the healthy quarter samples. In contrast, extremely low DNA yields insufficient for direct PCR amplification were recovered from the LSCC samples and all but one healthy quarter sample (4B at 399 ng/µl) with yields in the range of 0.3–2.6 ng/µl. A second round of DNA isolation returned similar results as confirmed by agarose gel electrophoresis (data not shown). To obtain sufficient amounts of DNA for downstream use, all samples (mastitis, healthy, and LSCC) were subsequently treated with the GenomiPhi V2 whole genomic DNA amplification system. One sample (1A) contained much higher amounts of DNA and was processed both with and without whole genome amplification to evaluate the effects of this treatment.

### Pyrosequencing Results

The sequencing run passed the quality control guidelines used by the DNA Facility at the University of Iowa with 1.5 million reads with an average read length of 305 nucleotides (367 median). After barcode sorting, 15,116–33,688 reads were obtained per sample with an average of 24,506 (±3,810) sequences per sample. Examining the number of reads returned by sample type showed that no sample type was disproportionately subjected to amplification during the sequencing run with an average of 23,755 (±4,994) reads for culture negative mastitis samples and 25,480 (±2,345) healthy and LSCC sample reads. After quality processing the sequences and using a 0.7 confidence cutoff for classification, an average of 2,364 (±4,220) sequences per mastitis sample and 4,016 (±3,060) sequences per healthy or LSCC samples were classified.

The number of sequences obtained, processed and classified for each sample can be found in Table S2. All the sequence data and analysis results are freely available at the project website http://www.microbiota.org/mastitis/.

### Comparison of Bacterial Community Compositions of Healthy and Non-culturable Clinical Mastitis Milk Samples

**Figure 1:**
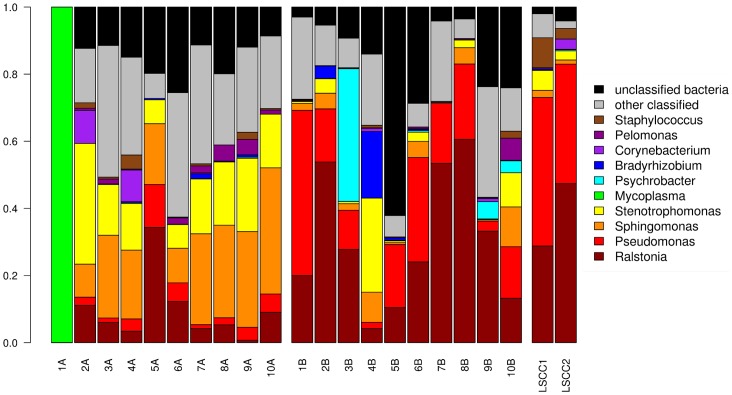
Taxonomic classifications for samples utilizing the RDP database. The normalized abundances of the top 10 most abundant bacterial genera determined using a RDP confidence threshold of 0.7 are shown. Sample pairs are labeled by animal (1–10) and clinical status as A (culture-negative clinical) or B (healthy), and the LSCC samples are labeled 1 and 2. Sample 1A (clinical) contained a known mastitis pathogen (*Mycoplasma* spp.) that was not detected in the healthy quarter sample 1B.

The average number of genera detected in clinical and healthy samples was 26.9 (±9.9) and 30.4 (±6.2), respectively. The number of operational taxonomic units (OTUs) present in each sample type was 48.7 (±19.1) in clinical samples and 72.6 (±35) in healthy samples. Examination of the top ten most abundant genera of the 16S rDNA taxonomic classifications for individual samples showed differences between the microbial communities of quarters of different states of health taken from the same animal (sample pairs) as well as general differences between all quarters of different states of health (clinical versus healthy) (Figure 1). The mastitic sample from one pair (1A) was observed to contain sequences predominantly classified as *Mycoplasma* spp. although this was not detected in any other samples.

**Figure 2:**
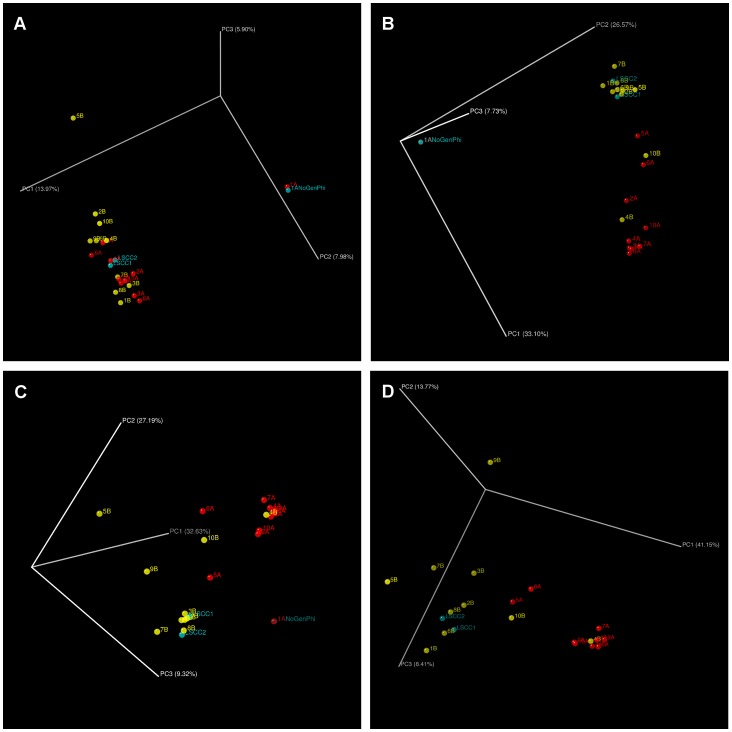
UniFrac PCoA images including non-amplified control. These images were captured from 3D UniFrac PCoA to illustrate differences in the microbiota among the different milk samples. The following UniFrac PCoA analyses were based on the OTU data, with only the first three principal coordinates shown. A) unweighted UniFrac with PC1 = 13.97%, PC2 = 7.98%, and PC3 = 5.90% (p = 0.083). B) weighted, normalized UniFrac with PC1 = 33.1%, PC2 = 26.57%, and PC3 = 7.73% (p = 0.001). C) weighted, non-normalized UniFrac with PC1 = 32.63%, PC2 = 27.19%, and PC3 = 9.32% (p = 0.001). D) weighted, non-normalized UniFrac, 1A excluded, with PC1 = 41.15%, PC2 = 13.77%, and PC3 = 8.41% (p = 0.001). The clustering observed between the culture negative clinical mastitis (red) and healthy (yellow) quarter milk samples indicates differences in the microbial compositions of these samples. The two LSCC samples (blue) cluster among other healthy samples. One sample not subjected to whole genome amplification (blue, 1A no GenomiPhi V2 amplification treatment) clusters tightly with the same sample subjected to whole genome amplification treatment (1A). In panels A–C the 1A clinical samples contribute to the greatest degree of observed dissimilarity likely due to its composition of predominantly *Mycoplasma* spp., as observed during taxonomic analysis.

**Figure 3:**
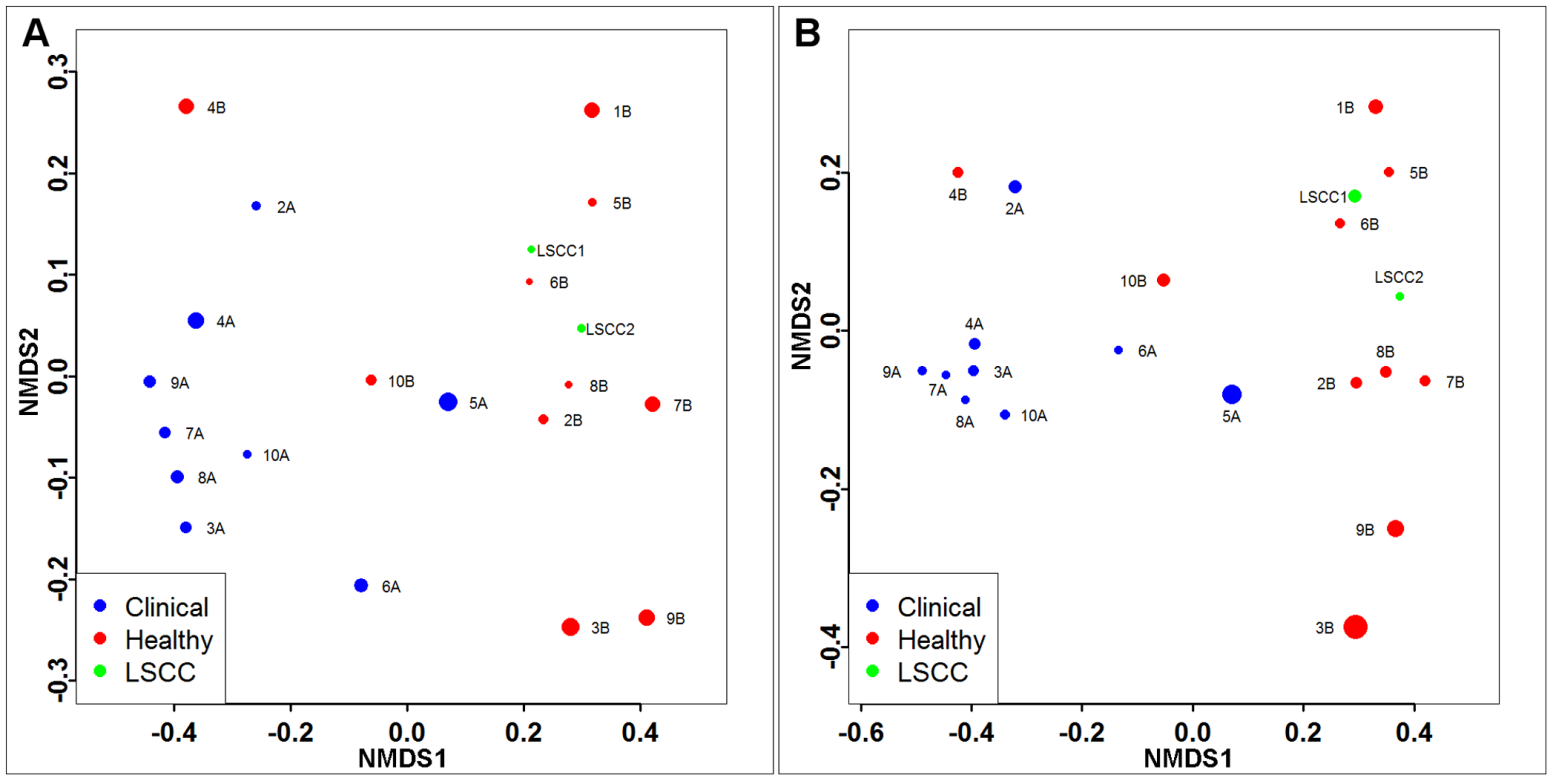
Bray-Curtis dissimilarity based non-metric multidimensional scaling. Using Bray-Curtis dissimilarity values, all samples, excluding 1A, were plotted using NMDS models. A) Healthy quarter and LSCC samples are observed to be more dissimilar to mastitis samples than each other with the exceptions of 4B and 5A. B) Samples plotted based on only the seven genera (see text) identified by univariate analysis. Using only these genera results in the similar delineation of clinical and non-clinical samples.

Differences in the microbiota of healthy versus culture negative clinical samples were also observed with phylogenetic beta-diversity calculations using UniFrac to plot principal coordinates analysis (PCoA) (Figure 2). In addition to healthy and clinical samples generally clustering separately, samples obtained from healthy animals (LSCC1-2) clustered with the healthy quarter samples included in the study. Separation of the mastitis and healthy samples within the PCoA plots are most easily visualized by viewing the PC2 and PC3 axes while PC1, representing the greatest variance among the samples, primarily illustrates the difference between sample 1A and the other samples (Figure 2). This relationship was maintained in both unweighted and weighted UniFrac regardless of normalization or whole genome amplification treatment. Discrete clustering of non-culturable clinical mastitis and healthy samples was more sharply defined in weighted UniFrac analyses except for samples 4B and 5A. The significantly different bacterial communities between the sample groups can also be seen by non-metric multidimensional scaling (NMDS) analysis using Bray-Curtis dissimilarity (Figure 3A) (perMANOVA test, p = 0.001; ANOSIM, p = 0.003). To improve visualization of the relationships among the samples, sample 1A was excluded due to the distortion of sample visualization as evidenced by NMDS plotting (Figure S1). The patterns of sample clustering within Figure 3 strongly resembled the separation previously observed in the PCoA figures even though two different beta distances measurements were used (UniFrac in Figure 2, and Bray-Curtis in Figure 3), thereby reinforcing our observation that there are robust differences in healthy and culture negative clinical microbiota.

To further evaluate differences in composition, all samples were compared using Fisher’s exact test and Wilcoxon signed rank tests on genus level classifications that were normalized for each sample by taking the number of sequences classified for each genus and dividing it by the total number of sequences classified at the bacterial domain level for the respective sample. The Fisher’s exact test did not indicate any significant differences in genus composition between samples, meaning there was no detectable significant bias in the presence/absence of any genera in the different sample types. However, the Wilcoxon signed rank test for normalized genus classifications indicated significantly higher (p<0.05, no multiple test correction) abundances of *Brevundimonas, Burkholderia*, *Sphingomonas*, and *Stenotrophomonas* in clinical samples and *Pseudomonas*, *Psychrobacter*, and *Ralstonia* in healthy samples (Table 1). Additional abundances and test results for the forty most abundant genera in clinical and healthy samples are listed in Table S3.

**Table 1:** Significant results for univariate analyses of genera classifications between sample pairs.

	Clinical Mean (%)	Healthy Mean (%)	Fisher’s ExactTest (p-value)	Wilcoxon signed ranktest (p-value)	Greater Relative Abundance
*Brevundimonas*	0.3321	0.1306	1	0.042315	Clinical
*Burkholderia*	1.1822	0.2823	1	0.019531	Clinical
*Pseudomonas*	3.8485	18.7531	1	0.009766	Healthy
*Psychrobacter*	0.0704	4.9304	0.069779	0.032969	Healthy
*Ralstonia*	8.6317	30.0565	1	0.027344	Healthy
*Sphingomonas*	20.4212	4.0238	1	0.003906	Clinical
*Stenotrophomonas*	15.2148	4.9642	1	0.027344	Clinical

Mean abundance calculations were performed for each sample using counts normalized by the total bacterial domain classified sequences for each sample.

Bray-Curtis dissimilarity values were also calculated based on the data from these seven genera and used to generate NMDS plots (Figure 3B) resulting in delineation of clinical and non-clinical samples similar to that seen in NMDS plots based on all data (Figure 3A).

## Discussion

Here we report the use of 16S rRNA gene diversity profiling to characterize the microbiota associated with milk from cows with mastitis for which the etiology is indeterminable by routine culturing techniques. By careful screening of milk samples from multiple dairy farms, we were able to select pairs of samples for 16S rRNA gene analysis that included both culture-negative mastitic and non-mastitic microbiota from the same animal. For comparison, we also included milk samples from visibly healthy animals with low somatic cell count milk. The results reveal new insights into how disease is linked to changes in the bacterial composition of milk and suggest significant roles for bacteria commonly found in the environment in mammary health and disease.

During DNA preparation, we observed that culture negative clinical milk samples, along with a sample from a single healthy quarter, yielded more DNA with overall higher viscosity than samples from healthy quarters. This was likely the result of elevated numbers of somatic cells in the clinical mastitis samples, which is a common feature of milk from diseased quarters [11]. Since the non-clinical milk samples did not yield sufficient quantities of DNA for further processing, we used whole genome amplification (WGA) (Materials and Methods) on all of the samples. In an attempt to evaluate the effect of WGA on our assessment of community composition, one sample with adequate amounts of DNA was prepared for amplicon sequencing both with and without WGA (1A). The WGA treatment was found to minimally affect this sample in terms of taxonomic composition and alpha-diversity (data not shown) as well as beta-diversity as visualized with PCoA (Figure 2). Unfortunately this sample was predominantly comprised of *Mycoplasma* classified sequences, which limits its utility in determining WGA skewing of the microbiota though it does demonstrate limited artifact introduction and population shift by the treatment for this sample at least. WGA has been used in other metagenomic studies [12]–[16], and we used an enzyme system shown to minimize amplification bias [17]. To further minimize the effect of any bias introduced by WGA, all samples were subjected to amplification, an approach that has been utilized in other studies [12]–[14], [18]. As this study focused on examining differences between samples as opposed to emphasizing detection of shared microbiota, any such bias should not affect the comparative analysis.

Analysis of the composition of the microbiota from the mastitic and non-mastitic quarter milk samples from the same animals revealed the presence of a large diversity of bacterial species present, even though no bacteria were detected by culture techniques. In addition, differences in microbial composition of the samples were observed. Such differences were apparent in taxonomic composition and reflected in beta-diversity measurements as illustrated in the PCoA analysis of the UniFrac distance and the NMDS analysis of the Bray-Curtis dissimilarity (Figures 2 and 3, respectively). The UniFrac PCoA showed that non-clinical and clinical samples generally fell within separate clusters based on clinical status. This result shows that the differences between samples from the same animal are based on the clinical status of each quarter and not on the variability between individual animals. The OTU diversity of all samples, except for 1A, was much greater than expected for culture-negative milk samples, especially for those obtained from quarters not exhibiting clinical signs of mastitis, as well as the LSCC samples from healthy animals. This likely reflects the relatively low abundance of many bacterial species in milk, as well as unique growth requirements that prevent their detection by standard culture methods.

Sample 1A, as indicated in the taxonomic analysis, was unique in that it contained predominantly *Mycoplasma* spp. at the genus level. This sample, independent of WGA, accounted for the greatest degree of variance in comparison to the rest of the samples. This result is consistent with the etiology of mastitis, however, as *M. bovis* is associated with this disease in cattle [19]. *M. bovis* is highly infectious between cattle, and infected herds can be identified using specific culturing methods or PCR [20]–[22], although identification of other *Mycoplasma* spp. can be difficult [22]–[25]. Out of the three dairies included in the study, *Mycoplasma* had previously been detected at only one location. The sporadic presence of non-typeable *Mycoplasma* spp. from individual clinical samples and bulk tank culture had been detected on that farm. The general absence of this genus from all but one sample further confirms that these herds have very little evidence of contagious *M. bovis* infection.

BLAST comparison of the denoised, trimmed sequence data from this sample to the Ribosomal Database Project (RDP) and NCBI -nr databases indicated that this isolate is most likely *M. californicum* (99.95% identity match, data not shown). Interestingly, *M. californicum* has also previously been associated with bovine mastitis and exhibits a biochemical profile very similar to that observed for *M. bovis* [26]–[29]. It is unclear if the decreased taxonomic diversity in sample 1A was the result of niche displacement of commensal organisms or an overabundance of *Mycoplasma* DNA that led to comparatively low sequences from other OTUs. Given the apparent abundance of *Mycoplasma* in sample 1A, we favor the explanation that this microorganism resulted in a displacement of the original bacterial microbiota. This result suggests that further research is needed to determine how bacterial pathogens may influence the “normal” microbiota of the mammary system.

Inspection of the data also showed that the select taxonomic profile of 5A more closely resembled that of healthy milk samples (Figure 1). However, the relatively high abundance of DNA isolated from this sample, and the lower number of classified sequences in comparison to the healthy sample from the same animal is consistent with patterns seen for other mastitis/healthy milk sample pairs. Similarly, the “healthy” sample 4B consistently clustered with mastitis samples although no clinical signs were observed in this quarter at the time of milk collection. As seen with 5A however, the abundance of DNA isolated from 4B more closely resembled yields from mastitis samples, including 4A, and the number of classifiable bacterial reads for 4A and B were consistent with profiles seen for other pairs (Table S2). It is possible this quarter would have developed clinical signs soon after the sample was taken.

The relative abundances of seven genera were found to be significantly different between the clinical and healthy samples with greater abundances of *Brevundimonas, Burkholderia*, *Sphingomonas*, and *Stenotrophomonas* found in clinical samples and *Pseudomonas*, *Psychrobacter*, and *Ralstonia* in healthy samples (Table 1). Interestingly, three of these genera (*Pseudomonas*, *Ralstonia*, and *Sphingomonas*) were also found to be among the 15 most abundant healthy human milk samples obtained from 16 healthy subjects at three different times [30]. Six abundant bacterial genera common to both studies include: *Bradyrhizobium, Corynebacterium, Pseudomonas*, *Ralstonia, Sphingomonas*, and *Staphylococcus*. Although significant differences in the relative abundances of *Bradyrhizobium*, *Corynebacterium* and *Staphylococcus* between clinical and healthy milk samples were not observed, their overall abundance and the number of abundant genera common to both studies illustrates a great degree of similarity between the two environments that may warrant further investigation. Similarly, some of the genera of interest detected in this study, most noticeably *Corynebacterium and Staphylococcus,* were also identified in a study of bacteria associated with the teat skin that sequenced cloned 16S rRNA genes obtained through culture dependent and independent methods [31]. Taking into account the proximity of the teat skin to the mucosal surface of the canal and our collection methods that preclude collection surface contact and collection of initial milk streams, it seems there is supporting evidence for overlap of the microbiota of these environments. This knowledge could be taken into account in future mastitis studies as microbial shifts of the teat surface may subsequently prove to be of interest as well.

Conversely only *Staphylococcus* was found to be abundant here and in another recent bovine mastitis study by Oikonomou *et al.* [32]. The high degree of similarity between this study and Hunt *et al*. (2011) versus Oikonomou *et al*. (2012) is likely a reflection of differences in identification of culture-negative samples, DNA isolation, and sequence classification methodologies. Their inclusion of samples our methods may not have classified as culture-negative, the differences in sample processing for DNA isolation, and finally, the utilization of a different database and sequence classification methodologies likely account for the dissimilarities between these two studies. Whereas the high degree of similarity with the human milk study may be partially explained as that study also used Mothur and only classified reads correctly aligning to the SILVA database. This may serve as an example to future comprehensive comparative analyses that before substantial comparisons can be made across studies, uniform processes must be utilized to reduce the amount of inherent experimental variability as acknowledged in the experimental design of The Human Microbiome Project [33].

Various *Burkholderia* spp. have been previously associated with infections in susceptible human populations, with *B. pseudomallei* documented as causing mastitis [34]–[36]. *B. cepacia* has been identified as causing subclinical mastitis in sheep as well as infections in other domestic animals [37]. The increased association of *Burkholderia* with mastitic quarter samples is consistent with these observations, although none was detected during milk sample culturing. *Brevundimonas* has also previously been detected in milk and was detected at low discrimination levels in conjunction with *Mannheimia haemolytica* in one study of subclinical mastitis [38], [39].

The finding of *Pseudomonas*, *Ralstonia*, and *Psychrobacter* at higher levels within normal healthy udders is intriguing. *Pseudomonas aeruginosa* is a well-known cause of mastitis in dairy cattle, associated with moderate to severe cases exhibiting obvious clinical signs, and it is typically readily grown and identified using standard milk culture methods. Thus, it is likely that the bacteria identified in the healthy samples are not *P. aeruginosa* but rather another *Pseudomonas* sp. that is less readily cultivated *in vitro*. To our knowledge, no species of *Ralstonia* or *Psychrobacter* have been ever confirmed as a cause of mastitis in dairy cattle. However, *Pseudomonas* and *Ralstonia* have been associated with contamination of water, including purified water systems [40], [41]. This association with water could represent a potential source of colonization of mammary tissues in cattle since modern milking practices rely heavily on water for sanitation of the milking units. Mastitis caused by *Pseudomonas aeruginosa* has been associated with contamination of water systems and teat disinfectants in the milking parlor [42]. It is therefore possible that a less pathogenic species of *Pseudomonas* had colonized the udders from water sources.

A previous study also identified *Pseudomonas* spp. to be associated with spoilage of dairy products [43]. *Ralstonia* spp. have been increasingly identified in ultra-high purity water systems and can withstand adverse environmental conditions that many bacteria cannot survive [40], [44]–[47]. These bacteria, therefore, are likely found in the milking environment (e.g., the milking machine system) where they can first come into contact with the teat and subsequently enter into the udder through the milking process and have been previously been detected in milk and cheese [48]. *Psychrobacter* spp. are found in a variety of environmental conditions and have been known to cause opportunistic disease in humans [49]–[51]. These bacteria have not been previously associated with mastitis but have been detected in raw milk and dairy environments [48], [52].

*Sphingomonas* and *Stenotrophomonas* were also identified as being more predominant in clinical mastitis quarters (Figure 1). Both have been previously detected in dairy environments in France [52]. *Stenotrophomonas maltophilia* has been reported in association with an outbreak of clinical mastitis in cattle in Japan [53]. In that study the bacteria were readily cultured from milk using a method very similar to that used for this work, suggesting that the *Stenotrophomonas* sequences identified here may be from a species other than *S. maltophilia*. It is also interesting to note that a *Stenotrophomonas* isolate has been shown to be involved in keratin degradation [54]. Given that a major innate immune mechanism of the bovine mammary gland is the production of a keratin plug covering the teat canal, the ability of a microorganism to degrade keratin would likely enhance its ability to colonize mammary tissues [55]. *Sphingomonas* spp. are unusual gram-negative bacteria replacing lipopolysaccharide (LPS) with glycosphingolipids (GSL), and have the ability to grow in a wide range of environments that are not tolerated by most other bacteria [56]–[59]. One study surveying microorganisms present in dairy production plants following disinfection detected an unidentified *Sphingomonas sp.* [60]. Also, *Sphingomonas paucimobilis* has been linked to a variety of nosocomial infections in humans [61], [62]. *S. paucimobilis* and *S. maltophilia* were both detected in a dairy study performed over the course of six years that examined gram-negative bacteria in milk samples with elevated somatic cell counts [63].

These results demonstrate that there are significant differences in the bacterial populations in milk from quarters showing signs of clinical mastitis in comparison to milk from healthy quarters, even though both sources were culture negative. While the biological significance of these findings requires further investigation, this study suggests new hypotheses to test. For example, the mastitis associated with culture negative samples could be attributed to small numbers of toxigenic bacteria that, while below limits of detection by culture-based methods, are sufficient to cause tissue inflammation. Alternatively, the changes in microbiota could predispose the quarters to disease by other etiological agents, such as viruses, fungi, or eukaryotic microorganisms. Conversely, the changes in the microbiota could be solely in response to inflammation caused exclusively by other unidentified pathogens.

The results presented here reveal that significant changes in the microbiota are found in milk from diseased quarters that cannot be detected by standard culture methods. This observation suggests that it may be possible to develop and apply new, more sensitive biomarkers at the sub-clinical level for early detection of the onset of clinical mastitis. A time course study would enable us to identify fluctuations in the milk microbiota, potentially revealing the most ideal microorganisms to track during disease progression. This study confirms the hypothesis that different microbial populations exist in culture negative mastitis cases and demonstrates the value of using metagenomic approaches to address questions of animal health and veterinary medicine.

Finally, the methods employed here to identify the bacterial genera associated with different disease states, demonstrates the usefulness of implementing advanced computational analyses and statistics in conjunction with 16S rDNA data. Although a larger time course study would likely better identify any bacteria correlated to disease onset and resolution, our results demonstrate that it may be possible to obtain such knowledge. Therefore, the application of these methods could direct the focus of future studies on heretofore poorly characterized microorganisms of interest. Such knowledge could enable targeted studies to develop customized probes and tests for use in preventative and early disease treatment benefiting both cows and producers.

## Materials and Methods

### Milk Collection and Bacterial Growth Testing

Milk sample collection protocols were approved by the Iowa State University Institutional Animal Care and Use Committee prior to initiation of the study (IACUC #6-09-6762-B). Samples were collected from three local dairy farms, including the Iowa State University Dairy Research Facility. The farms are all free-stall operations milking Holstein dairy cattle in Iowa. All were in commercial milk production, milked in parlors, and fed a total mixed ration formulated by a bovine dairy nutritionist. Rolling herd averages for all farms are over 20,000 pounds of milk in a 305 day lactation. Individual cows with mastitis were identified by animal care personnel during normal milking preparation. Cows with clinical mastitis were assigned a mastitis score of 1–3 based on the severity of mastitis (1 = abnormal milk alone, 2 = abnormal milk with local signs of inflammation in the mammary system and 3 = abnormal milk and systemic signs of illness). Clinical samples selected for DNA sequencing were required to have a score of 1 or greater. Sterile milk cultures were collected after sanitization of the quarter following standard recommendations by the National Mastitis Council’s Laboratory Handbook on Bovine Mastitis [64]. Briefly, teats were dipped in iodine followed by physical scrubbing with alcohol. Following surface cleaning, several streams of foremilk were then removed prior to sample collection. Concurrently, a sterile milk sample was collected from an unaffected quarter of the same cow. All sample pairs were immediately refrigerated and transported to the College of Veterinary Medicine at Iowa State University and were processed.

Two ml aliquots of each milk sample was transferred into a sterile vial and frozen until DNA isolation. All milk samples were directly cultured for aerobic bacteria using standard culture techniques described [64], which included pipetting 0.1 ml of milk from clinical mastitis and normal samples onto trypticase soy agar plates with 5% bovine blood (BAP). In addition, milk from clinical mastitis samples was used to inoculate MacConkey agar plates. Plates were incubated aerobically at 37°C for up to 48 hours. All milk samples from quarters with clinical mastitis also underwent an enrichment culture process. Two milliliters of enrichment media was inoculated with an equal volume of milk from the clinical mastitis sample and incubated in a water bath at 37°C for 4 hours. At the conclusion of the enrichment process 0.1 ml of the enriched sample was spread onto BAP and MacConkey and plates were incubated aerobically.

After 24 and 48 hours of incubation, all plates were inspected for growth and all growth was identified using standard techniques [64], and results were reported back to the dairy farms. Samples from quarters with clinical mastitis that had no colony growth after 48 hours were identified as “No Growth”. Ten of these sample pairs were selected for partial 16S rRNA gene analysis in addition to two LSCC samples from healthy cows that were collected and tested after initial collection of samples for the study.

### DNA Isolation and Preparation for Pyrosequencing

Genomic DNA from milk was purified from paired samples (culture negative mastitis and control quarters from the same cow) originating from 10 cows exhibiting clinical signs of mastitis and two individual samples from healthy individuals (Table S1) using the Qiagen DNA Mini Kit (Valencia, CA) and the Blood or Body Fluid Spin Protocol to process 400 µl of each sample with the following modifications: all vortexing was limited to ten seconds of pulse vortexing, 100 µL of elution buffer was used, and elution was carried out following a 5 minute incubation of the columns with elution buffer at room-temperature. DNA preparations were then quantified using Quan-iT™ PicoGreen® dsDNA Kit (Invitrogen, Carlsbad, CA, USA) and a Nanodrop 3300 Fluorospectrometer (Thermo Fisher Scientific, Wilmington, DE, USA).

Each genomic DNA sample was subjected to whole genome DNA amplification (Illustra™ GenomiPhi™ V2 DNA Amplification Kit, GE Health Sciences, Piscataway, NJ, USA) following the manufacturer’s standard protocol. This amplification was carried out in duplicate and reaction products were pooled post-heat inactivation. The resulting DNA was purified using ethanol-precipitation and resuspended in Qiagen AE elution buffer [65]. These and an unamplified mastitis DNA control sample were then quantified using a Nanodrop 1000 Spectrophotometer (Thermo Fisher Scientific, Wilmington, DE, USA) and diluted to 100 ng/µl. The resulting products were then used as template, in addition to one non-GenomiPhi amplified sample that already contained sufficient bacterial DNA, for PCR reactions to generate 16S rDNA amplicon libraries. Primer sequences (Table S4) from 5′ to 3′, included the Roche 454 Life Sciences® Titanium fusion Primers A or B (required for 454 sequencing), a multiplex identifier sequence (MID), and sequences corresponding to the BSF8 or BSR357 primers used to amplify the V1–2 region of bacterial 16S rRNA genes [66] (Roche technical bulletins 013-2009 and 005-2009). All primers were synthesized and HPLC purified by Integrated DNA Technologies (Coralville, IA, USA).

PCR reactions were carried out in triplicate, and included negative controls, in 50 µl volumes using the Phusion® High-Fidelity PCR Master Mix with HF buffer (New England Biolabs, Ipswich, MA, USA), 2 µM each primer, and 100 ng DNA template on a BioRad MJ Mini Personal Thermal Cycler (BioRad, Hercules, CA, USA). The thermal cycler program was as follows: 98°C for 3 minutes; followed by thirty cycles of 98°C for 30 seconds, 55°C for 30 seconds, and 72°C for 30 seconds; and finished with 72°C for 10 minutes and a 4°C hold. The sizes of all PCR products were confirmed by agarose gel electrophoresis on 1% SB buffer gels (Faster Better Media LLC, Hunt Valley, MD, USA). After confirming all reactions and negative controls were satisfactory, the three PCR reactions per sample were pooled and purified with 0.7×Agencourt® AMPureXP® beads (Beckman Coulter Inc., Brea, CA, USA) and eluted in 50 µl TE buffer. This DNA was then re-purified using a 1.6×AMPureXP bead concentration and eluted with 25 µl of TE into DNA LoBind 1.5 ml tubes (Eppendorf, Hauppauge, NY, USA) (Roche Amplicon Library Preparation Method Manual, GS FLX Titanium Series, October 2009). Products were then quantified with the Nanodrop 1000 Spectrophotometer and diluted to 1 ng/µl. The quality of the 16S rDNA amplicon libraries was tested by running them on a 2100 Agilent bioanalyzer on a DNA High Sensitivity chip (Iowa State University DNA Facility, Ames, IA). Samples were submitted to the University of Iowa DNA Facility for Roche 454 GS FLX Titanium chemistry pyrosequencing as two pools on one plate. Each pool contained 16S rDNA amplicon libraries prepared from five pairs of samples from culture-negative clinical and non-clinical quarters from the same animal and one culture-negative, LSCC milk sample; one pool also included the non-GenomiPhi amplified library from a non-clinical sample.

### Sequence Processing and OTU Assignment

Sequence handling and analysis were carried out following the Mothur curation pipeline v1.0c [67]. Briefly, fasta, quality and flow files were extracted from Roche files from each pool and flowgrams were trimmed and denoised (minflows = 360, maxflows = 720, pdiffs = 0, bdiffs = 0). Fasta files were processed by identifying perfect matches to primer and barcode sequences in the reads or the reverse complement sequences, trimming these sequences, and sequences meeting the 200 nucleotide minimum length requirement were output (pdiffs = 0, bdiffs = 0, maxhomop = 8, minlength = 200, flip = T). The number of unique sequences was also determined at this and subsequent steps in the analysis. After concatenating the read output from the two pools, the sequences or their reverse-complement were aligned to the SILVA database [68]. Sequences not aligning within the optimized alignment region were removed from the analysis with the screening function (optimize = start-end, minlength = 250, criteria = 90). Chimeric sequences were identified using chimera.uchime in Mothur and removed [69]. After generating distance matrices from aligned sequences and clustering OTUs using a distance of 0.03, taxonomic assignments were made using the RDP classifier v2.4 trained on dataset 7 with a confidence threshold of 0.7 at genus level and 0.9 at the domain level [5]. Cyanobacteria were removed as environmental contaminants.

### Statistical Analyses

Customized R scripts were implemented to evaluate the significance of differences observed for each genus or OTU between clinical and healthy samples. Fisher’s exact and Wilcoxon signed rank tests were applied to each pair of clinical and healthy samples using normalized counts for each genus or OTU [70]. Normalized counts were obtained for each sample by dividing the number of sequences classified for each genus (or OTU) by the total number of sequences classified at the bacterial domain level for that sample. Beta-diversity visualizations using weighted and unweighted UniFrac PCoA were performed using OTU counts for the samples [71]–[74]. Using the R statistical package ecodist, Bray-Curtis dissimilarities were calculated for each pair and group of sample types and used in NMDS [75]. Bray-Curtis based analysis of similarity (ANOSIM) and perMANOVA [76] were implemented with the R package *vegan* [77].

## Supporting Information

Figure S1 **Bray-Curtis dissimilarity based non-metric multidimensional scaling.** Using Bray-Curtis dissimilarity values, samples were plotted using NMDS models. Due to the high dissimilarity associated with sample 1A, this sample was omitted from subsequent analyses to improve visualization of the relationships among other samples.
(GZ) Click here for additional data file.

Table S1 Additional information about the samples used for sequencing. Information includes animal identification, dairy farm, date of sample collection, days in milk, health status, severity score and culture results.
(GZ) Click here for additional data file.

Table S2 DNA sequence processing results.
(GZ) Click here for additional data file.

Table S3 Univariate analyses of the 40 most abundant genera classifications between samples.
(GZ) Click here for additional data file.

Table S4 Sequences of primers used in the study.
(GZ) Click here for additional data file.
